# Therapeutic ultrasound combined with microbubbles improves atherosclerotic plaque stability by selectively destroying the intraplaque neovasculature

**DOI:** 10.7150/thno.39553

**Published:** 2020-01-22

**Authors:** Xinzhong Li, Shengcun Guo, Tong Xu, Xiang He, Yili Sun, Xiaoqiang Chen, Shiping Cao, Xiaoyun Si, Wangjun Liao, Yulin Liao, Yuan Han, Jianping Bin

**Affiliations:** 1 Department of Cardiology, State Key Laboratory of Organ Failure Research, Nanfang Hospital, Southern Medical University, Guangzhou, China.; 2 Guangzhou Regenerative Medicine and Health Guangdong Laboratory, 510005 Guangzhou, China.; 3 Department of Cardiology, The First Affiliated Hospital of Zhengzhou University, Zhengzhou, China.; 4 Department of Oncology, Nanfang Hospital, Southern Medical University, Guangzhou, China.

**Keywords:** therapeutic ultrasound, microbubbles, atherosclerosis, angiogenesis, plaque stability.

## Abstract

**Objective**: The current antiangiogenic therapy for atherosclerotic plaques was mainly achieved by the use of antiangiogenic drugs, but serious side effects have limited the clinical application. The present study investigated whether therapeutic ultrasound (TUS) treatment with appropriate pressure could selectively deplete the neovasculature in vulnerable plaques to improve its stability with no side effects on the body; the underlying mechanisms were also explored.

**Methods and Results**: A mouse model of advanced atherosclerosis was generated by maintaining apolipoprotein E-deficient (ApoE-/-) mice on a hypercholesterolemic diet (HCD). Plaque, skeletal muscle, mesentery and skin tissue from 114 atheroma-bearing mice were subjected to sham therapy, an ultrasound application combined with microbubbles at four different ultrasound pressures (1.0, 2.0, 3.0, 5.0 MPa), or ultrasound at 5.0 MPa alone. Microvessel density (MVD) was assessed by immunofluorescence and immunohistochemical methods. The plaque necrotic center/fiber cap (NC/FC) ratio and vulnerability index were calculated to evaluate plaque vulnerability. Twenty-four hours after TUS treatment at 3.0 MPa, the MVD in the plaque was substantially decreased by 84% (p < 0.05), while there was almost no change in MVD and neovessel density (NVD) in normal tissues, including skeletal muscle, mesentery and skin. Additionally, a marked reduction in the number of immature vessels was observed in the plaques (reduced by 90%, p < 0.05), whereas the number of mature vessels was not significantly decreased. Furthermore, TUS treatment at 3.0 MPa significantly improved plaque stability, as reflected by the NC/FC ratio and vulnerability index, which may be due to the selective destruction of intraplaque neovascularization by TUS treatment, thereby decreasing the extravasation of erythrocytes and leading to vascular inflammation alleviation and thin-cap fibroatheroma reduction.

**Conclusions**: TUS treatment at 3.0 MPa selectively depleted plaque neovessels and improved the stability of vulnerable plaques through a reduction in erythrocyte extravasation and inflammatory mediator influx, with no significant effect on normal tissue.

## Introduction

Atherosclerotic plaque neovascularization, a conduit for erythrocyte leakage and subsequent inflammatory response induction, is implicated in plaque instability and atheroma progression, which causes the majority of acute cardiovascular events 1, 2. Antiangiogenesis treatment may constitute a valuable therapeutic strategy for atherosclerotic plaque stabilization 3. Currently, the inhibition of vulnerable plaque neovascularization is mainly achieved through the application of antiangiogenic drugs targeting different aspects of angiogenesis, such as blocking the synthesis and release of angiogenic factors, inhibiting endothelial function, and modulating endothelial degradation of the surrounding matrix 4-6. However, a range of associated adverse effects, including the inhibition of physiological neovascularization and increase in the risk of thrombosis, heart failure, hypertension, proteinuria and renal insufficiency 7, 8, have largely impeded the introduction of antiangiogenic drugs into clinical practice. In addition, ultrasound-targeted microbubble destruction (UTMD), which facilitates drug or gene targeted delivery, has been applied to the treatment of atherosclerotic plaques 9-11. While, there are still some serious defects such as missing suitable clinical delivery system and uncertain therapeutic potencies, which limit the promotion and clinical application of UTMD to some extent 12, 13.

Recently, therapeutic ultrasound (TUS) treatment has gained attention as a novel antineovascular intervention that mainly exerts antivascular effects through inertial cavitation (IC) 14. The forceful collapse of intravenous microbubbles gives rise to transient microjets that produce a mechanical force on the vessel wall, leading to vessel injury or even rupture 15, 16. In contrast to mature vessels in normal tissues, which are not affected, the structural incompleteness of the neovasculature increases its susceptibility to TUS treatment 17. In tumor therapy, TUS has been shown to selectively destroy neovascularization, which markedly decreases tumor perfusion and improves the long-term prognosis 18. Pathological neovascularization is a concomitant feature of atherosclerotic plaque progression: it promotes plaque progression by facilitating the extravasation of erythrocytes and influx of inflammatory mediators 19. Emerging evidence suggests that more than 80% of the neovasculature in atherosclerotic plaques is immature, characterized by large intercellular spaces, incomplete basement membranes and the absence of pericyte-mediated structural integrity protection, making them more sensitive than normal blood vessels to mechanical damage caused by ultrasound-mediated cavitation effects 20. In addition, it is worth mentioning that US-MB approaches could be used to monitor intraplaque angiogenesis and evaluate the physiological consequences of therapeutic interventions 21-23. More importantly, as a critical physical medium, microbubbles flow smoothly into the neovasculature and attach to the endothelium 24, allowing them to mediate acoustic cavitation, especially IC elicited by US, causing damage to the neovasculature. Therefore, US-MB treatment may hold potential as a new therapeutic paradigm for atherosclerotic plaques. However, whether US-MB-mediated cavitation, which avoids the systemic side effects of drug use, can destroy neovasculature in atherosclerotic plaques and improve plaque stability has not been confirmed.

In the present study, we hypothesized that TUS treatment with appropriate pressure could selectively deplete the plaque neovasculature in advanced atherosclerosis, resulting in improved plaque stability. Moreover, we investigated the underlying mechanism of TUS-induced stabilization of vulnerable plaques via the destruction of the neovasculature. Our results indicate that TUS will be an effective strategy for stabilizing vulnerable plaques through antivascular therapy.

## Materials and Methods

### Animal models and experimental protocol

The animal study protocol was approved by the Animal Research Committee at Southern Medical University, and all procedures were performed in accordance with the NIH Guide for the Care and Use of Laboratory Animals 25.

Male apolipoprotein E-deficient (ApoE^-/-^) mice (6 weeks of age) were originally purchased from the Vital River Laboratory Animal Technology Co., Ltd. (Beijing, China), and were provided with a standard diet (Oriental Yeast) and tap water ad libitum until required for experiments. For experiments, 8-week-old mice (n=114) were fed a HCD (containing 21% fat and 0.15% cholesterol by weight) for up to 30 weeks. Then, the mice were randomly assigned to receive ultrasound (US)-MB/US treatment.

To validate the correlation of plaque neovascularization with its instability, 6 mice were randomly selected from the 114 ApoE^-/-^ HCD-fed mice as the control group. These animals were sacrificed using an overdose of anesthesia (150 mg/kg pentobarbital sodium injected intraperitoneally), and the abdominal aorta was collected for hematoxylin and eosin (H&E), immunohistochemical staining and immunofluorescence.

To determine the appropriate level of US pressure, the remaining 108 HCD-fed ApoE^-/-^ mice were randomly divided into a sham group and five treatment groups (n=18 per group) for experimentation on their abdominal aortas. The abdomen was covered with a foam pad while the abdominal aorta was insonated. The sham treatment was performed with the therapeutic US transducer in the off setting. The five treatment groups were divided into four US-MB treatment groups and a US treatment group. The TUS treatment groups were insonated with different acoustic pressures (1.0, 2.0, 3.0, and 5.0 MPa) and injected with microbubbles, while the US treatment group was insonated with 5.0 MPa US in the absence of microbubbles. These mice were sacrificed before treatment, immediately after treatment (0 h) and 24 h after treatment. Abdominal aortas were collected for immunohistochemistry (IHC).

To explore the mechanism by which plaque instability is reversed, 30 HCD-fed ApoE^-/-^ mice were randomly selected for TUS treatment with appropriate ultrasound pressure that had been previously determined. H&E, immunohistochemical staining and double-labeling immunofluorescence were used on the abdominal aorta before and 0 h, 24 h, 8 weeks and 16 weeks after US-MB treatment.

**Figure 1:**
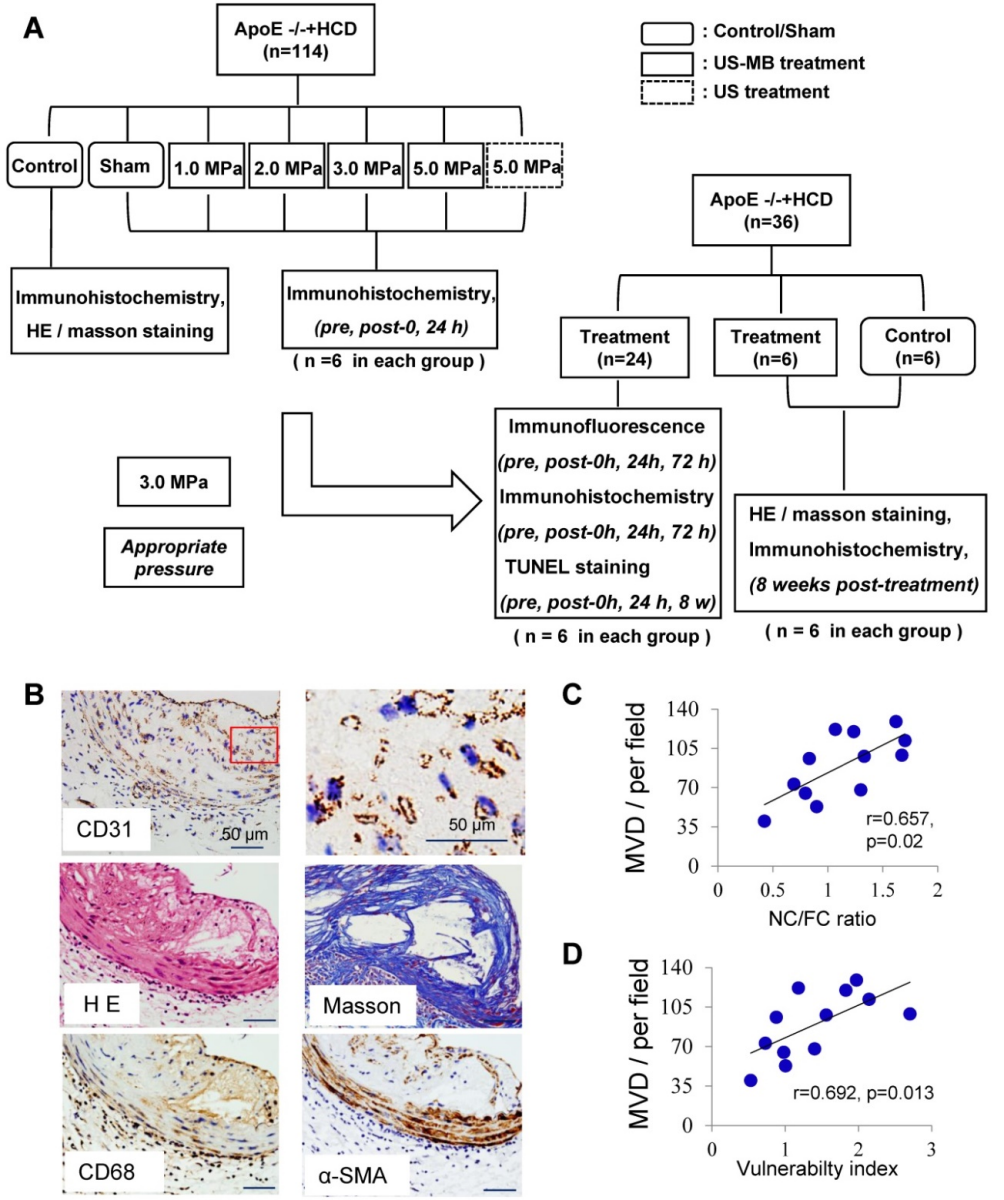
** Plaque vessel density is associated with plaque vulnerability.** (A) Illustration of the experimental protocol. (B) Representative images of aortic tissue as visualized by H&E and Masson's trichrome staining or labeled with antibodies against CD31, α-SMA and CD68 (bars, 50 μm). Correlations between MVD of the plaque, (C) plaque vulnerability index and (D) plaque NC/FC.

To evaluate the therapeutic effect of TUS treatment on plaque instability, 12 HCD-fed ApoE^-/-^ mice were randomly divided into TUS treatment (n=6) and control (n=6) groups; the treatment group was insonated with appropriate US pressure, while the control group was not insonated. Abdominal aortic plaque stability was detected with H&E/Masson's trichrome staining and IHC at 8 weeks after US-MB treatment. The experimental protocol is illustrated in Fig. 1A.

### Therapeutic US system

A pulsed therapeutic US device with a KHT-017 transducer (DCT-700, Shenzhen Well. D Medical Electronic, Shenzhen, China) was employed as the therapeutic US source. The transducer has a diameter of 2.0 cm and is operated with a frequency of 1.0 MHz; the peak negative pressure ranges from 0.5 to 5.0 MPa and the duty cycle from 0.1% to 20%. A needle hydrophone (HNP-0400, Onda, USA) adjusted by a precision 3-D motion stage was set up to measure the acoustic output of the transducer at a range of 0.3-6.0 cm outside the front surface. The sound pressure value during treatment was based on the display on the instrument panel. To maintain the position of the transducer in the process of TUS treatment, the transducer was fixed on a steel stand with a scale and held 3 mm over the abdominal skin. Subsequently, the tail vein was cannulated for microbubble injection, and the US gel was placed over the abdominal skin to ensure direct contact between the transducer and the abdominal skin. The TUS treatment was performed on the abdominal aorta after intravenous injection of 1.5 × 10^8^ microbubbles in a total volume of 0.1 ml; the US treatment was performed without microbubbles. For the *in vitro* experiment, the endothelial cells were arranged in a single layer at the bottom of a 30-mm diameter petri dish filled with DMEM. Then, as described previously 26, the petri dish was inverted into a 100 mm petri dish and then filled with DMEM, sterile saline containing 1.5 × 10^8^ microbubbles was injected into the medium through a syringe and homemade pillow. The transducer was disinfected with 70% alcohol and then placed vertically against the dish with the aid of coupling gel. The transducer was operated at a frequency of 1 MHz with a pulse repetition frequency of 10 Hz and a duty cycle of 0.19%. The acoustic pressure output could be switched between 1.0 MPa and 5.0 MPa. The treatment was performed with an intermittent mode of 2 seconds on and 8 seconds off for 30 seconds. The cavitation generated by MBs is represented by two-dimensional imaging in the water in Fig. S1A, B.

### MB preparation and characterization

The MBs were generated as previously described 16. The concentration size and distribution of MBs were analyzed using a coulter counter (Multisizer III, Beckman Coulter, FL, USA), which was presented in Fig. S1C, D. The structure of MBs was visualized by a microscope (BX51; Olympus, Tokyo, Japan). The *in vitro* cavitation of MBs was determined by the therapeutic US system described above. B-mode images were acquired before and after treatment by microbubble-enhanced ultrasound (MEUS).

### Histology and immunohistology

After the mice were sacrificed, tissues of the abdominal aorta were fixed in 4% paraformaldehyde and embedded in paraffin. Serial 3-μm thick paraffin sections were cut and stained with H&E, Masson's trichrome staining and immunostaining for the observation of histological changes as described previously 27.

To evaluate plaque vulnerability, the plaque necrotic center/fiber cap (NC/FC) ratio and vulnerability index were calculated as described previously. H&E and Masson's trichrome (MST-8003; Matxin Labs Pvt., Ltd., Bangalore, India) were used to measure the area of lipid deposition and collagen fiber content. Sections were incubated using a rabbit polyclonal against mouse α-smooth muscle actin (α-SMA) and cluster of differentiation (CD) 68 (all from Abcam, Cambridge, MA, USA) to stain for smooth muscle cells (SMCs) and macrophages, respectively. The positive staining areas of SMCs, macrophages, lipid deposition and collagen were quantified using Image-Pro Plus (IPP, Media Cybernetics, Rockville, MD, USA) by two individuals who were blinded to the experimental design and are expressed as the percentage of positive-to-total plaque area. The NC/FC ratio was measured as the ratio of lipid deposition to collagen fiber, and the plaque vulnerability index was calculated using the following formula: vulnerability index = (macrophages %+ lipids %) / (SMCs %+ collagen %).

To evaluate the microvessel density (MVD), sections were also subjected to immunostaining with an anti-CD31 antibody (Abcam, Cambridge, MA, USA) to label endothelial cells. Microvessels were identified as channels surrounded by a layer of endothelial cells. Density counts of microvessels were calculated as CD31-positive endothelial cells with or without a lumen in 5 randomly selected high-power (400×) fields from 6 separate sections of each sample. The MVD was quantified by two individuals who were blinded to the experimental design and is expressed as the average number of microvessels per field.

To explore the mechanism of the US-MB treatment-induced reversal of plaque instability, sections were stained with an anti-TER-119 antibody (BD Biosciences, San Jose, CA) to label erythrocytes leaking from the vessels in the plaque; immunostaining with anti-CD68 and antivascular cell adhesion molecule-1 (VCAM-1) antibodies was used to label macrophages and adhesion molecules, respectively. Extravasation of erythrocytes in the plaques was quantified by counting the average number of erythrocytes in 10 randomly selected 400× high-power fields. The positive staining area of macrophages and adhesion molecules in the plaque was quantified as described above.

### Confocal immunofluorescence microscopy

To quantitatively analyze plaque angiogenesis, sections were subjected to double-labeling immunofluorescence as described previously 18. A rabbit polyclonal antibody against mouse CD31 (Abcam, Cambridge, MA, USA) and a rat polyclonal antibody against mouse α-SMA or SM22α (Abcam, Cambridge, MA, USA) were used to detect endotheliocytes and pericytes, respectively. Nuclei were counterstained with DAPI (Sigma-Aldrich, St. Louis, MO). HBG2 (Recombinant Human Hemoglobin Gamma G) (prospec, Belgium) was used to stimulate endothelial cells according to the manufacturer's instructions. Images were evaluated by a confocal fluorescence microscope (Carl Zeiss, LSM880) and were merged using IPP software. A neovessel was defined as a CD31-positive microvessel rarely surrounded by mural pericytes and positive for α-SMA.

### Scratch wound (migration) assay

Evaluation of endothelial cell migration ability was performed by a scratch wound assay as described previously 28. Treated HUVECs were cultured in endothelial basal medium (EBM)-2 at 37°C and 5% CO_2_. Scratches in the cell monolayer were generated with a 200-µL tip, and the cells were imaged at 0, 6, 24 and 48 h with a Zeiss Axiovert 135 microscope. Subsequently, the distance between cell fronts was measured with an AxioVision documentation system (Zeiss).

### Tube-formation and spheroid-formation assays

Treated cells were performed as previously described 28. Matrigel Growth Factor Reduced (BD) Basement Membrane Matrix was prepared and incubated with 1.5×10^4^ HUVECs in EBM-2 and 1% fetal calf serum (FCS) for 4 h. After the cells were fixed with 4% PFA, images of tube formation were obtained on a Zeiss AxioVision microscope (Jena, Germany). The cumulative length of all sprouts of each spheroid or the maximal distance of the migrated cells were used to quantify HUVEC spheroids. Approximately 10 spheroids were analyzed for each experiment.

### Flow cytometry

Apoptosis was analyzed by flow cytometry according to the instructions of the Annexin V/PI kit as described previously 28. After digestion in trypsin without EDTA, cells in single-cell suspensions at a density of 1×10^6^/mL were treated with US-MB for 30 seconds, and the percentage of apoptotic cells was determined in triplicate (%).

### Western blot analysis

Western blot analysis was performed as previously reported 29. The primary antibodies used were against MCP-1 (1:100, ab157808; Abcam), VCAM-1 (1:200, sc-374429; Santa Cruz) and MMP-2 (1:50, ab178681; Abcam). β-Actin (1:200, sc-25778; Santa Cruz) was used as a loading control. Alexa Fluor 480 (1:10,000, ab175772; Abcam) was used as the secondary antibody. Immunoreactive bands were visualized with Odyssey Software (version 1.2; LI-COR, Lincoln, NE, USA). Protein expression was measured using ImageJ analysis software (NIH, Bethesda, MD, USA).

### TUNEL

TUNEL staining was performed as previously described 30. The abdominal aorta tissue were fixed with paraformaldehyde for 1 hour, and an *in situ* cell death detection kit (TMR red) (Roche, Switzerland) were used to label apoptic cells. Nuclei were counterstained with DAPI (Sigma-Aldrich, St. Louis, MO). Six visual fields were selected from each group to calculate the proportion of TUNEL-positive cells. Apoptotic cells were compared and analyzed by the percentages (TUNEL staining positive cells / DAPI staining positive cells). Images were collected by confocal fluorescence microscope (Carl Zeiss, LSM880).

### Statistical analysis

Data were analyzed using SPSS v.13.0 (SPSS, Inc., Chicago, IL, USA) and are presented as the mean ± standard deviation. Comparisons between two groups were performed with the independent samples t-test. Comparisons between different treatment groups or time points were performed using a one-way analysis of variance. A Bonferroni correction was calculated for multiple comparisons of continuous variables with equal variance (determined by the homogeneity of variance test), while Dunnett's T3 test was used for multiple comparisons without equal variance. Spearman's rank correlation was used to assess the linear correlation between selected variables. A p value <0.05 was considered statistically significant.

## Results

### Plaque neovascularization is associated with plaque vulnerability

CD31 expression in abdominal aortic plaque was detected to assess the increased density of microvessels in ApoE^-/-^+HCD mice (Fig. 1B); H&E and Masson's trichrome staining, as well as α-SMA and CD68 immunostaining, was used to assess the plaque NC/FC and its vulnerability index. Notably, the MVD in the whole plaque was correlated with plaque indicators such as the plaque vulnerability index and NC/FC (Fig. 1C, 1D).

**Figure 2:**
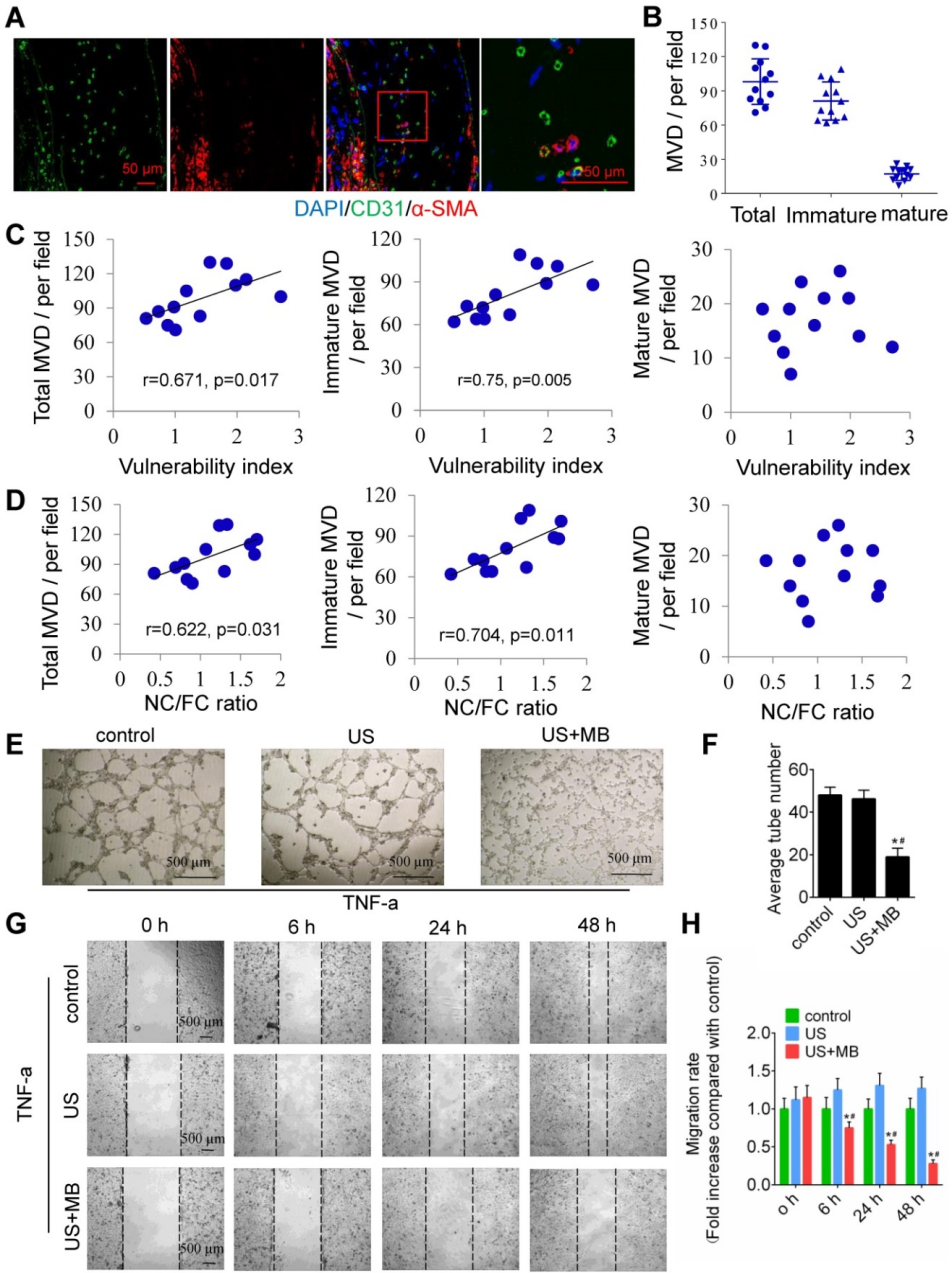
** Neovascularization in the plaque causes instability, and TUS-MB destroys the neovasculature *in vitro*.** (A) Representative images of confocal immunofluorescence staining of CD31 and α-SMA (bars, 50 μm). (B) Quantitative analyses of the total, immature and mature MVDs. MVD, microvessel density. (C) Correlations between plaque vulnerability index and the total MVD, immature MVD, and mature MVD. (D) Correlations between plaque NC/FC ratio and total MVD, immature MVD, and mature MVD. (E) Destruction of neovasculature by TUS-MB *in vitro*. Sonicated HUVECs with an ultrasonic pressure of 1.0 MPa were seeded onto a Matrigel matrix and stimulated with TNF-α (10 ng/ml). The average number of tubes formed per field was statistically analyzed (bars, 500 μm). (F) Quantification of the average number of tubes. *p<0.05 vs. control. #p<0.05 vs. US; n=6 per group. (G) Cell migration was assessed using wound-healing assays. Images were captured at 0, 6, 24, and 48 h after TNF-α stimulation (10 ng/mL) and TUS-MB treatment. The horizontal line indicates the wound edge. Migration was estimated by measuring cell numbers within the wound region (bars, 500 μm). (H) Quantification of the endothelial cell migration rate. *p<0.05 vs. control. #p<0.05 vs. TNF-α; n=6 per group.

To test the relationship between plaque neovascularization and vulnerability, confocal immunofluorescence staining of CD31 and α-SMA was used. The results showed that a majority of plaque microvessels lacked pericytes (82.67%), which indicates an immature state, also known as neovascularization (Fig. 2A, 2B). Interestingly, both the total and immature MVDs in the whole plaques were correlated with plaque indicators such as the plaque vulnerability index and the NC/FC ratio, whereas the mature MVD was not related to the plaque vulnerability index or NC/FC ratio (Fig. 2C, 2D).

In addition, US-MB could destroy neovascularization *in vitro*. A schematic diagram of the *in vitro* experiment is shown in Fig. S2A. US-MB at 1.0 MPa significantly decreased tumor necrosis factor (TNF)-α-induced tube formation (p<0.05; Fig. 2E, F). Increased expression of TNF-α contributes to endothelial cell migration, thereby affecting vascular stability. We found that US-MB at 1.0 MPa markedly decreased the number of migrating cells induced by TNF-α (p<0.05; Fig. 2G, H). Moreover, flow cytometry results showed that, compared with control or ultrasound, US-MB at 1.0 MPa significantly promoted endothelial cell apoptosis (p<0.05; Fig. S2B). Transwell and spheroid-formation assays suggested that US-MB at 1.0 MPa markedly suppressed endothelial cell migration and vascular sprouting induced by VEGF (p<0.05; Fig. S2C, D).

### The effects of US-MB treatment on plaque, skin, mesentery and muscle tissue: Results for various pressures

**Figure 3:**
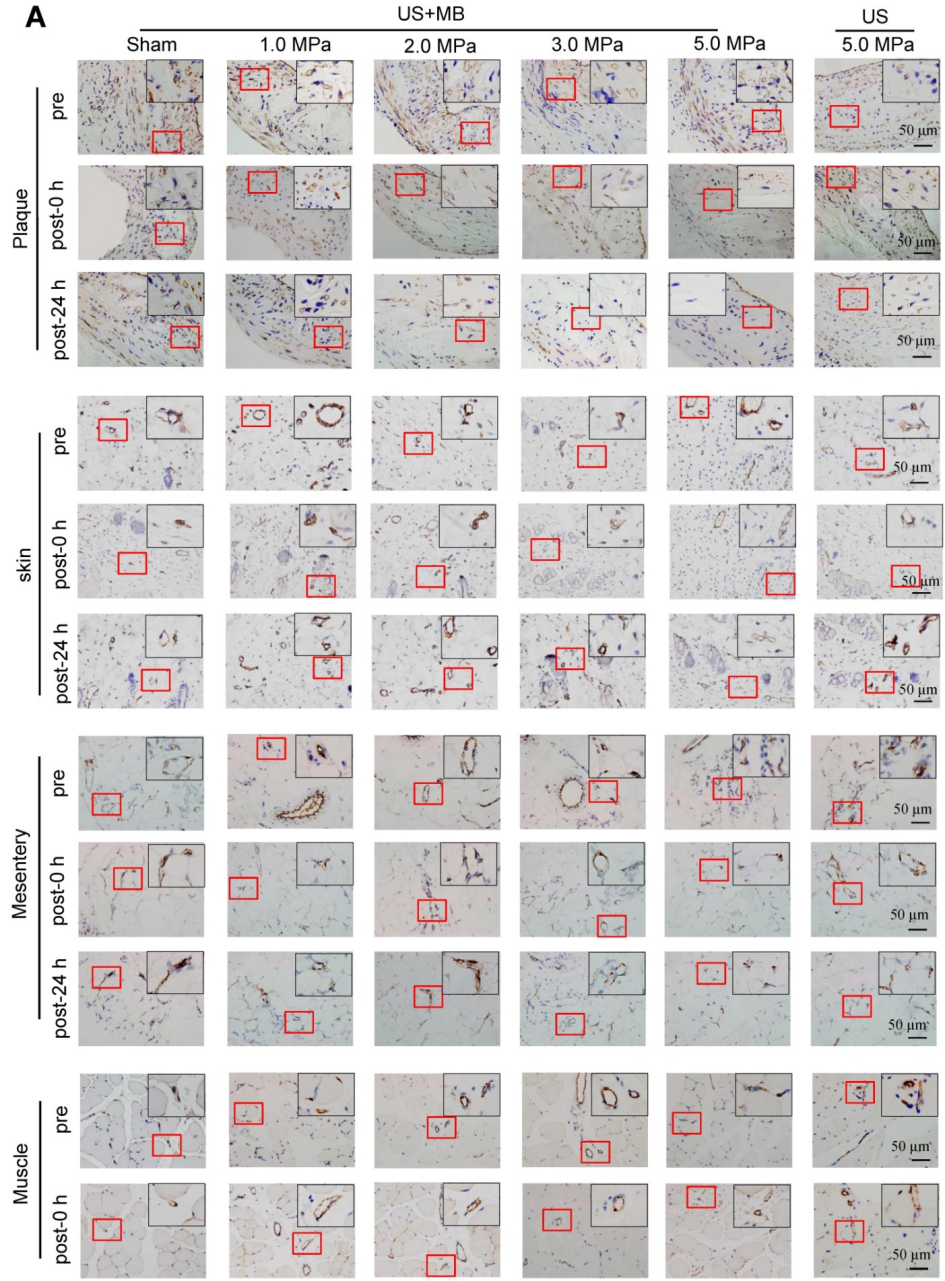

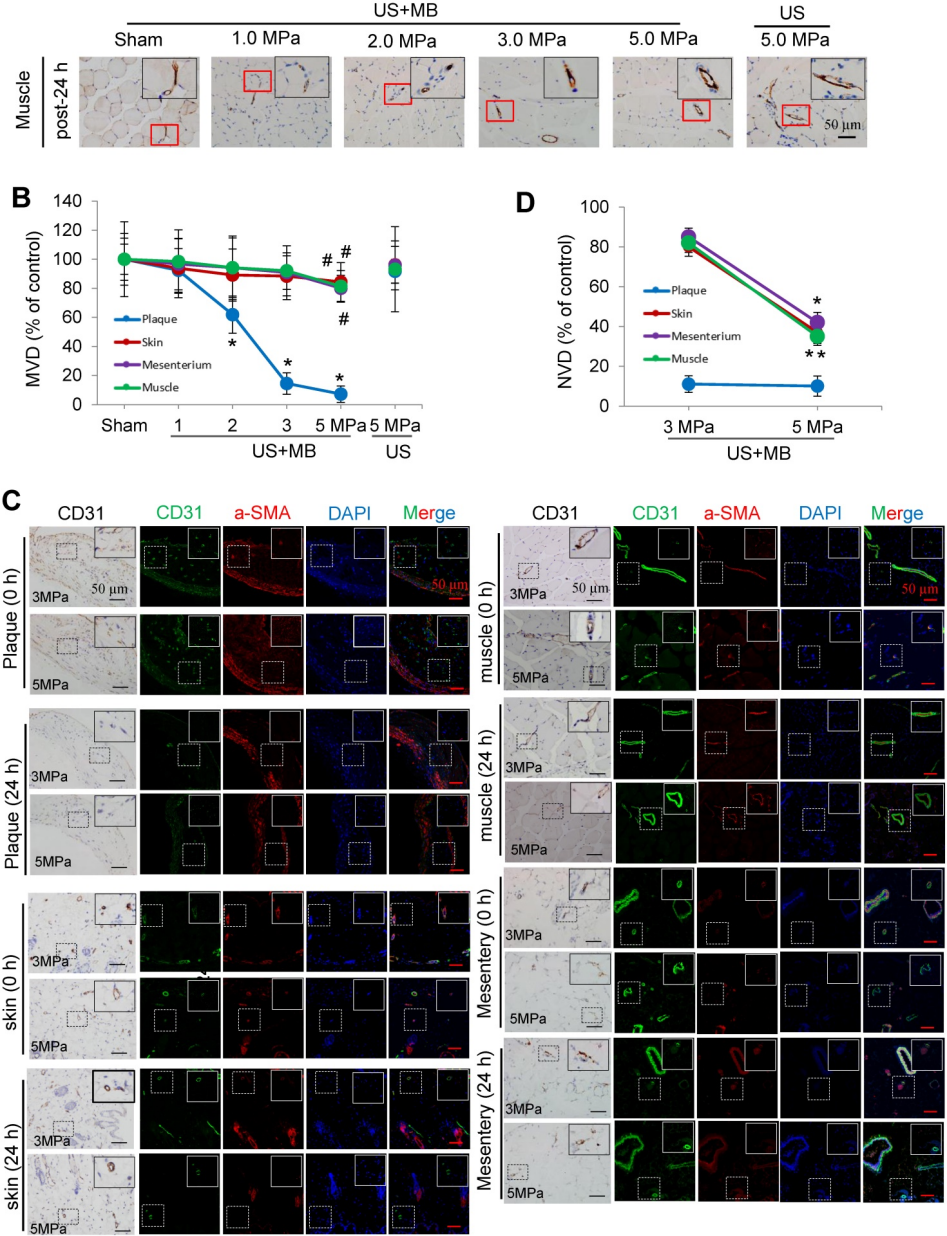
** Effects of US-MB treatment on microvessels in plaque, skin, mesentery and muscle: Results for various pressures.** (A) Representative images of immunohistochemical staining for the endothelial marker CD31 in plaque, skin, mesentery and muscle (bars, 50 μm). (B) Quantitative analysis of the MVD at 24 h after treatment. *p < 0.05, ^#^p < 0.05, vs. the respective sham groups. MVD, microvessel density. (C) Representative images of immunohistochemical staining for CD31 and confocal immunofluorescence of microvessels in plaque, skin, mesentery and muscle stained for CD31 (green) and α-SMA (red) at 0 h and 24 h after treatment (bars, 50 μm). (D) Quantitative analysis of neovessels. *p < 0.05 vs. post 0 h. SMA, smooth muscle actin.

CD31 IHC was used to reveal the MVD and structures in the plaque, skin, mesentery and muscle tissue in the different treatment groups immediately and 24 h after US-MB treatment. The heat map of the transducer sound pressure on the horizontal and vertical axes is shown in Fig. S3A-C, which suggested that the sound beam emitted was similar to a focal column and had almost no scattering within 6.0 cm. Before treatment, microvessels were mostly circular and clearly visible in the plaque, skin, mesentery and muscle in the 1.0, 2.0, 3.0, 5.0 MPa US-MB and 5.0 MPa US treatment groups. Immediately after treatment, the MVD in the plaque in each groups was similar to that in the sham group, while the structures of plaque microvessels in the 3.0 MPa and 5.0 MPa US-MB treatment groups were mostly incomplete, with vessel fragments scattered in the region, and microvessels in the skin, mesentery and muscle were still relatively shaped in each group (Fig. 3A). Twenty-four hours after treatment, there was a significant reduction in the MVD of the plaque in the 2.0, 3.0, and 5.0 MPa US-MB treatment groups compared with the sham group. The MVD of the plaque was reduced by approximately 86% and 92% in the 3.0 and 5.0 MPa groups, respectively (p < 0.05), and it was reduced by 38% in the 2.0 MPa US-MB treatment group (p < 0.05; Fig. 3A, 3B). In contrast, there was no significant reduction in the MVD of the skin, mesentery or muscle in the US-MB groups with 1.0, 2.0, 3.0 or 5.0 MPa and no significant reduction in the MVD of the plaque, skin, mesentery or muscle in the US treatment group compared with the sham group (Fig. 3A, 3B).

We further used confocal immunofluorescence staining to evaluate the effect of US-MB treatment with 3.0 MPa and 5.0 MPa on NVD. Both ultrasonic pressures significantly reduced the density of new blood vessels in the plaque. However, for the skin, mesentery and skeletal muscle, the 3.0 MPa treatment group showed little effect on the NVD, while the 5.0 MPa treatment group had a significantly reduced NVD (Fig. 3C, 3D). In addition, the results of H&E staining suggested that changes in microbubble concentration within a certain range had almost no effect on normal tissues (Fig. S4A).

### Selective depletion of plaque microvasculature based on vessel maturation

**Figure 4:**
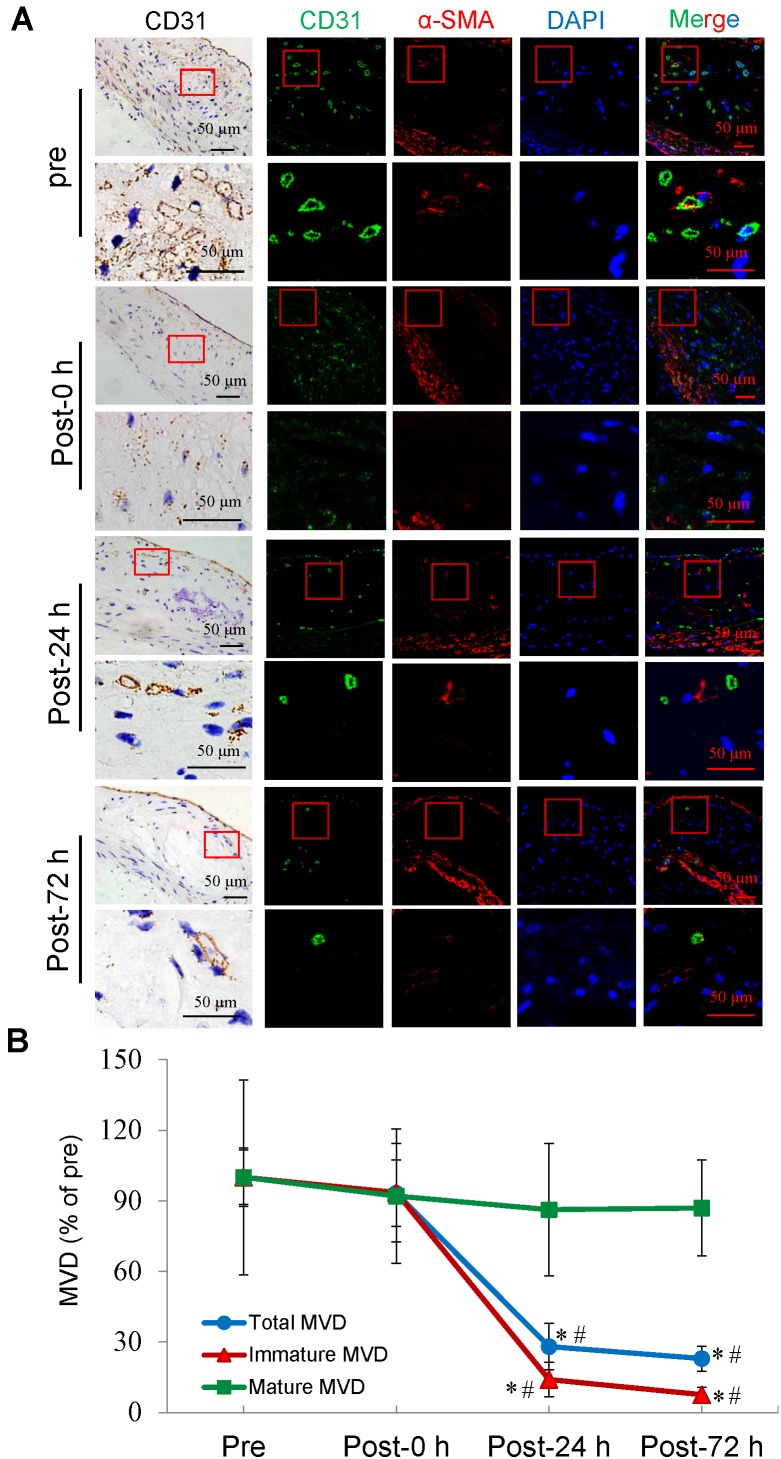
** Effects of US-MB treatment at 3.0 MPa on vessels at different maturation stages.** (A) Representative images of immunohistochemical staining for CD31 and confocal immunofluorescence of microvessels in plaques stained for CD31 (green) and α-SMA (red) at different treatment time points (bars, 50 μm). (B) Quantitative analysis of total, immature and mature microvessels. *p < 0.05 vs. pretreatment, ^#^p < 0.05 vs. post 0 h. SMA, smooth muscle actin.

Confocal immunofluorescence staining showed that the majority of plaque microvessels were immature. Immediately after US-MB treatment at 3.0 MPa, there was no reduction in the MVD of the total, immature, or mature microvessels. At 24 h after treatment, the total and the immature MVDs of the plaque were significantly reduced by approximately 72% and 86% (p<0.05). At 72 h after treatment, the number of total and immature microvessels were further significantly reduced by 77% and 92% (p<0.05), whereas there was no significant reduction in the number of mature vessels at different time points after treatment (Fig. 4A, 4B). These findings may demonstrate that selective depletion of the plaque microvasculature is based on vessel maturity.

### US-MB treatment reduces the extravasation of erythrocytes and the expression of macrophage markers and adhesion molecules

**Figure 5:**
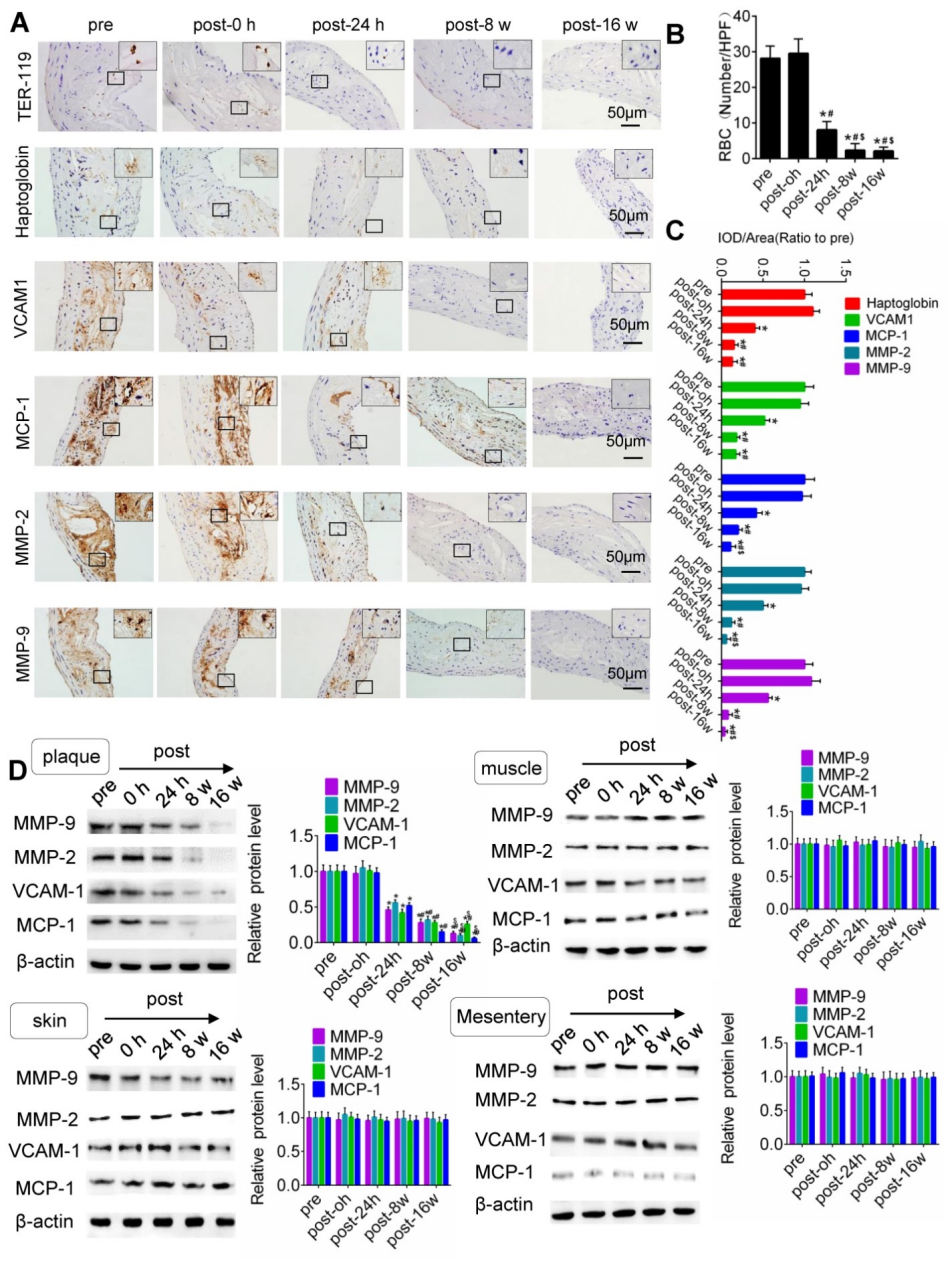
** TUS-MB treatment reduced intraplaque extravasation of erythrocytes and secondary inflammation.** (A) Representative images of immunohistochemical staining for TER-119, hemoglobin, VCAM1, MCP-1 and MMP-2 pretreatment and at 0 h, 24 h and 8 weeks after treatment with TUS-MB at 3.0 MPa (bars, 50 μm). (B) Quantification of the average number of red blood cells. *p<0.05 vs. pre. ^#^p<0.05 vs. post-24 h; n=6 per group. (C) Quantification of the expression of hemoglobin, VCAM1, MCP-1 and MMP-2 at different time points. (D) Representative immunoprecipitation images of VCAM1, MCP-1, MMP-2 and MMP-9 in plaque, skin, muscle and mesentery pretreatment and at 0 h, 24 h, 8 w and 16 w after treatment with TUS-MB at 3.0 MPa (bars, 50 μm). (E) Quantification of the immunoblot strip. *p<0.05 vs. pretreatment. ^#^p<0.05 vs. 24 h posttreatment; n=6 per group.

IHC results for TER-119, CD68, VCAM-1, MMP-2 and MMP-9 in the plaque is shown in Fig. 5. Compared with that pretreatment, immediately after US-MB treatment at 3.0 MPa, the extravasation of erythrocytes in the plaque was increased (p>0.05); in contrast, the extravasation of erythrocytes was significantly decreased at 24 h, 8 weeks and 16 weeks (p<0.05) after US-MB treatment (Fig. 5A, 5B). Overflowing haptoglobin induced inflammation (p<0.05; Fig. S5A, B). There was no significant difference in the expression of macrophage markers immediately after US-MB treatment compared with pretreatment; at 24 h, 8 and 16 weeks after treatment, the expression of macrophage markers was significantly decreased (p<0.05) (Fig. 5A, 5C). Furthermore, a similar expression pattern was observed in endothelial inflammation, fiber cap thickness and platelet activation, which were labeled by VCAM-1, MMP-2, MMP-9 and CD41 in the plaque (Fig. 5A, 5D and Fig. S5C).

### US-MB treatment reverses plaque instability

**Figure 6:**
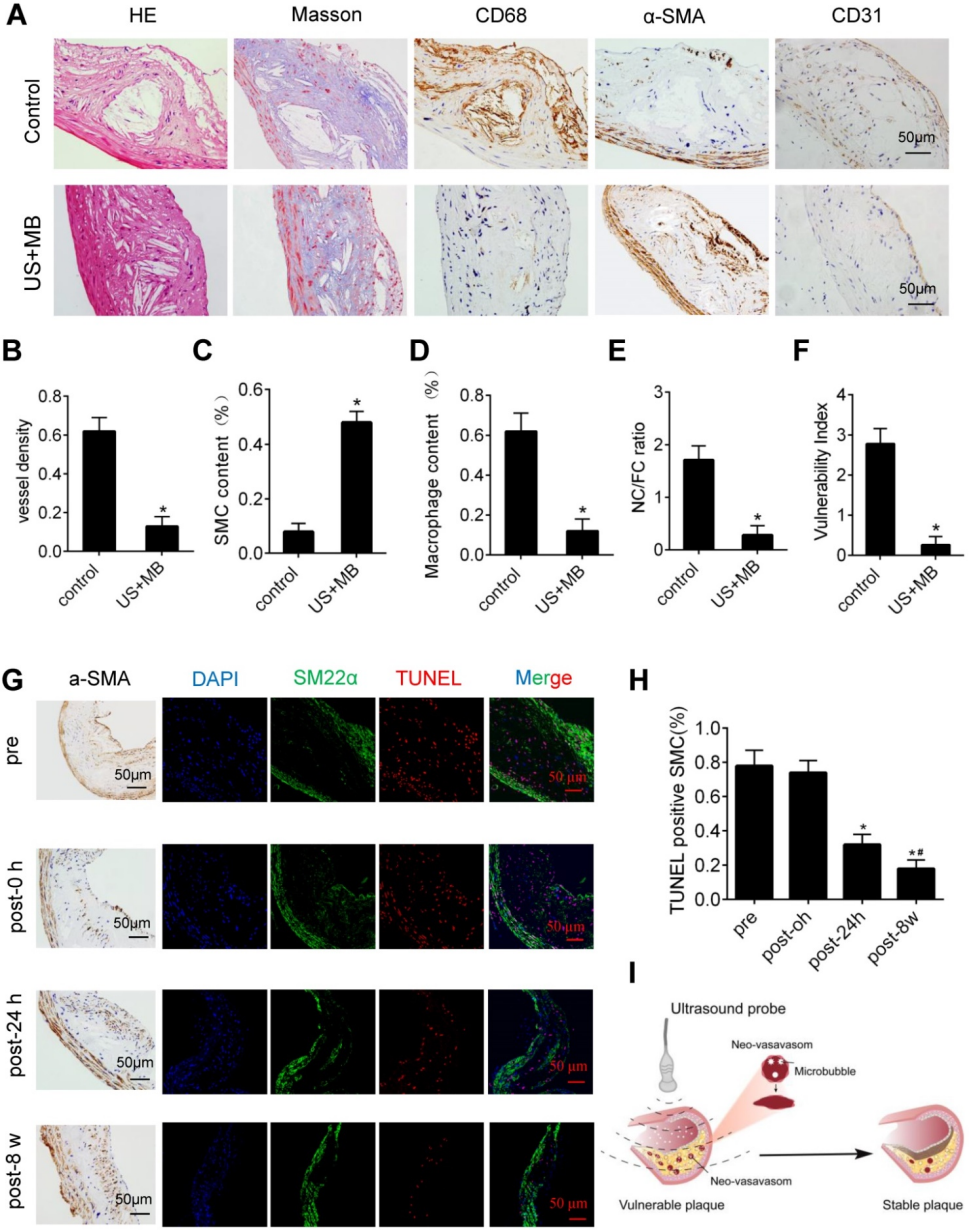
** Effect of US-MB treatment on plaque instability.** (A) Representative images of aortic tissue as visualized by H&E and Masson's trichrome staining or labeled with antibodies against α-SMA, CD68 and CD31 (bars, 50 μm). Quantitative analyses of (B) MVD density, (C) plaque macrophage and (D) SMC numbers are shown (n=6 per group). (D) Plaque NC/FC and (E) vulnerability index were calculated. *p<0.01 vs. control. SMA, smooth muscle actin. (F) Representative images of immunohistochemical staining for DAPI, SM22α and TUNEL (bars, 50 μm). (G) Quantitative analysis of apoptotic SMCs at different time points. *p < 0.05 vs. pretreatment, #p < 0.05 vs. 0 h posttreatment. SMA, smooth muscle actin. (H) Illustration of therapeutic ultrasound-microbubble (TUS-MB) stabilizing vulnerable plaque by damaging the neovascularization in plaque.

H&E and Masson's trichrome staining, as well as α-SMA, CD68 and CD31 immunolabeling of abdominal aortic plaques (Fig. 6A), showed that the microvessel density was markedly reduced, and the number of SMCs in plaques was significantly increased in the US-MB treatment groups compared with the control group (Fig. 6B, 6C). In addition, the number of macrophages was significantly decreased in the US-MB treatment group compared with the control group (Fig. 6D). Notably, the plaque NC/FC ratio and vulnerability index, which reflect plaque instability, were also significantly increased in the US-MB treatment groups compared with the control group (Fig. 6E, 6F). Additionally, TUNEL staining indicated that at 24 h as well as 8 weeks after US-MB treatment, fewer apoptotic SMCs were labeled (Fig. 6G, 6H), which is consistent with the increase in the number of SMCs mentioned above.

## Discussion

In the current study, we found that TUS treatment with an appropriate US pressure (3.0 MPa) destroyed intraplaque neovasculature and improved atherosclerotic plaque stability with no effect on normal tissues (Fig. 6I). The underlying mechanism may be the selective destruction of intraplaque neovascularization by TUS treatment to reduce the extravasation of erythrocytes, resulting in vascular inflammation alleviation and thin-cap fibroatheroma reduction, thereby enhancing plaque stability. This approach of ultrasound-driven microbubble cavitation, which selectively damages new blood vessels in plaque and avoids systemic side effects, may represent a novel strategy for stabilizing vulnerable atherosclerotic plaques.

It is generally believed that TUS at an appropriate US pressure can confer excellent antiangiogenesis efficacy and minor side effects. US pressure is one of the most important parameters that determine the inertial cavitation 31. To identify the US energy most suitable for destroying the intraplaque neovasculature, we designed and performed a series of comparative observations of *in vivo* vessels in plaque, skin, mesentery and muscle at four different levels of US pressure with or without microbubbles. Our results indicate that when treated with US-MB, the intraplaque neovascular density decreased gradually with an increase in ultrasonic pressure, reaching a plateau at 3.0 MPa, and did not further decrease even if the ultrasonic pressure was increased to 5.0 MPa. The vessel density in normal tissues, including skin, mesentery and muscle, was almost unaffected by US-MB treatment. This difference may be attributable to most of the blood vessels in the plaque being immature, whereas immature neovasculature in normal tissue accounts for only a small percentage of the vasculature 32. Our immunofluorescence results showed that the vast majority of plaque vessels were immature, in accordance with previous studies 33, 34. The immature vessels are known to be structurally abnormal and are characterized by increased endothelial gaps, defective basement membranes, and low rates of pericyte coverage, which accounts for their increased vulnerability to US-MB treatment 35. Furthermore, we compared the effects of US-MB treatment with 3.0 MPa or 5.0 MPa on normal tissue neovascularization and found that treatment with 5 MPa destroyed most of the neovasculature in normal tissue, while treatment at 3 MPa had little effect. One possible explanation for this phenomenon is that, compared with new vessels in the plaque, the neovasculature in normal tissues has better integrity due to relatively complete gap junctions between endothelial cells and can therefore resist the higher ultrasound pressure and survive 36, 37. Notably, our results showed that US-MB at a US pressure of 1.0 MPa destroyed approximately 80% of the forming tubes *in vitro*, the structure of which is similar to that of the neovasculature in plaque. This finding also indirectly indicates that intraplaque neovascularization is highly sensitive to cavitation.

Previous studies have shown that shear stress may induce rupture of the fibrous cap and lead to thrombosis 38, 39. Our present IHC staining for MMP-9, MMP-2 and CD41, which represented fiber cap thickness and platelet activation, did not increase after TUS treatment at 3.0 MPa and continued to decline over time. In addition, US pressure of 3.0 MPa with different MB concentrations had no effect on normal tissues. This suggested that TUS treatment at 3.0 MPa did not increase the risk of fibrous cap rupture and thrombosis. However, higher intensity ultrasound may induce these risks by destroying the microbubbles, which requires careful attention. The above results indicate that the US pressure of 3.0 MPa is appropriate and safe for antiangiogenesis treatment of plaques. Previous studies have shown a positive relationship between microbubble-mediated US cavitation intensity and vascular damage. Previously, we observed reduced vessel density in muscle and skin after US treatment at 5.0 MPa 18, however, in the present study, we used a lower duty cycle and microbubble concentration, which reduced the cavitation intensity and therefore had little effect on the vascular density in normal tissue. Therefore, as a novel antiangiogenic treatment strategy, TUS treatment at 3.0 MPa, selectively destroys the intraplaque neovasculature and has no adverse effects on normal tissue.

As a critical factor leading to plaque instability, neovascularization has long been considered a key target for the treatment of vulnerable plaques 40, 41. Accumulating evidence has shown that effective antineovascular therapy can stabilize vulnerable plaques and reduce the occurrence of secondary adverse events, which is mainly achieved through the use of antiangiogenic drugs, including those that block angiogenic factors, reduce matrix degradation and inhibit endothelial function 3, 42, 43. Additionally, microbubble-mediated ultrasound technology for aiding drug and gene delivery had been applied for neovascular destruction in plaque. However, such modalities were still limited with respect to optimization of delivery system and uncertain treatment effects. Moreover, the relevant studies lacked an exploration of the correlation between vascular damage and plaque stability, as well as the underlying mechanism. In the present study, we found that intraplaque neovasculature labeled with CD31 and α-SMA antibodies was positively associated with the plaque vulnerability index and NC/FC ratio, which indicated plaque instability. More importantly, the current results suggest that TUS can effectively destroy the intraplaque neovasculature. The intraplaque immunofluorescence results showed that TUS at a US pressure of 3.0 MPa significantly reduced neovascular density, as assessed by CD31 and α-SMA staining. More importantly, our results indicate that the destruction of the neovasculature by TUS significantly improved plaque stability. After treatment with TUS at a pressure of 3.0 MPa, the number of macrophages in the plaques was markedly reduced, the number of SMCs was obviously increased, and the corresponding vulnerability index and NC/FC ratio was decreased, which means that the stability of the plaque was effectively improved. Therefore, TUS can effectively destroy new blood vessels in plaques and improve the stability of vulnerable plaques.

Atherosclerotic plaque angiogenesis facilitates the extravasation of erythrocytes and inflammatory mediator influx, therefore causing an increased risk of secondary adverse events 44. Furthermore, a positive feedback loop could operate in atherosclerosis and chronic inflammation, whereby new vessels deliver inflammatory cells that further promote angiogenesis 45. Destruction of the plaque neovasculature by TUS, which reduces the extravasation of erythrocytes and levels of secondary inflammation mediators, could interrupt this cycle, thereby decreasing macrophage infiltration and metalloproteinase content. Our current IHC results showed that the number of extravasated red blood cells and hemoglobin at 24 h and 8 weeks after treatment was significantly reduced in the 3.0 MPa TUS group compared with the control group. Moreover, macrophage infiltration and the levels of metalloproteinases, which could digest the plaque fiber cap, causing plaque rupture, were markedly decreased. The same phenomenon was reflected in the immunoprecipitation data. We noted that the stability of vulnerable plaques, as reflected by the vulnerability index and NC/FC ratio, was significantly improved after treatment. A similar strategy for antiangiogenic therapy and erythrocyte leakage alleviation in plaque has been investigated in previous studies 46. However, these studies mainly interfered with neovascularization through drug therapy, which would cause serious side effects and limit their clinical application. The TUS treatment strategy in the current study could selectively destroy neovessels, thereby stabilizing plaques with no effects on normal tissue, and may be an ideal method to treat vulnerable plaques. It was meaningful that our previous study applied the TUS strategy to thrombolytic therapy in rat and canine models, which suggested that the current treatment for vulnerable plaques in mice also had potential for future use in humans but needed to be investigated by further clinical trials. It should be mentioned that it is difficult to completely damage intraplaque neovessels using a single session of TUS treatment. Consequently, residual plaque neovessels might still cause extravasation of erythrocytes, which could be a trigger of plaque vulnerability. Therefore, further research should be encouraged to explore whether multiple episodes of treatment can further destroy plaque angiogenesis and improve plaque stability.

There are several limitations to our study. First, the US probe used in this study is not yet accurately positioned to a local position. The application of more accurate ultrasound tools in the future will make it possible to avoid the potential harm caused by current TUS. Second, we focused on identifying an appropriate US pressure parameter; it will be preferable to use comparative investigations with intensive pressure gradients to explore the therapeutic window of US pressure. In addition, our current research was mainly carried out in mice, and although the sound pressure and illumination distance used in the present study have potential value for clinical applications, the effects of TUS treatment should be verified in large animals or humans in the future. Finally, the microbubbles in our present study were not targeted microbubbles. Although US has favorable time and spatial positioning, which facilitates the local destruction of blood vessels, for the clinical feasibility of TUS treatment, it might be preferable to develop neovascular-targeted microbubbles to achieve precise therapy.

In conclusion, TUS treatment at 3.0 MPa selectively depleted the plaque neovasculature, resulting in a substantial cessation of erythrocyte extravasation and influx of inflammatory mediators without a significant effect on normal tissue, consequently improving the stability of vulnerable plaques.

## Supplementary Material

Supplementary figures.

## Abbreviations

TUS: therapeutic ultrasound
MB: microbubble
MVD: microvessel density
NVD: neovessel density
IC: inertial cavitation
HCD: hypercholesterolemic diet
vWF: von Willebrand factor
ApoE-/-: apolipoprotein E-deficient
VCAM-1: vascular cell adhesion molecule-1
SMC: smooth muscle cell
PBS: phosphate-buffered saline
α-SMA: α-smooth muscle actin
NC/FC: necrotic center/fiber cap ratio
TNF-α: tumor necrosis factor α
HUVEC: human umbilical vein endothelial cells
RBC: red blood cells

## References

[1] Parma L, Baganha F, Quax PHA, de Vries MR (2017). Plaque angiogenesis and intraplaque hemorrhage in atherosclerosis. Eur J Pharmacol.

[2] de Vries MR, Quax PH (2016). Plaque angiogenesis and its relation to inflammation and atherosclerotic plaque destabilization. Curr Opin Lipidol.

[3] Moulton KS, Vakili K, Zurakowski D, Soliman M, Butterfield C, Sylvin E (2003). Inhibition of plaque neovascularization reduces macrophage accumulation and progression of advanced atherosclerosis. Proc Natl Acad Sci U S A.

[4] Moulton KS, Heller E, Konerding MA, Flynn E, Palinski W, Folkman J (1999). Angiogenesis inhibitors endostatin or TNP-470 reduce intimal neovascularization and plaque growth in apolipoprotein E-deficient mice. Circulation.

[5] Kampschulte M, Gunkel I, Stieger P, Sedding DG, Brinkmann A, Ritman EL (2014). Thalidomide influences atherogenesis in aortas of ApoE(-/-)/LDLR (-/-) double knockout mice: a nano-CT study. Int J Cardiovasc Imaging.

[6] Drinane M, Mollmark J, Zagorchev L, Moodie K, Sun B, Hall A (2009). The antiangiogenic activity of rPAI-1(23) inhibits vasa vasorum and growth of atherosclerotic plaque. Circ Res.

[7] Qi WX, Shen Z, Tang LN, Yao Y (2014). Congestive heart failure risk in cancer patients treated with vascular endothelial growth factor tyrosine kinase inhibitors: a systematic review and meta-analysis of 36 clinical trials. Br J Clin Pharmacol.

[8] Ferroni P, Formica V, Roselli M, Guadagni F (2010). Thromboembolic events in patients treated with anti-angiogenic drugs. Curr Vasc Pharmacol.

[9] Yuan H, Hu H, Sun J, Shi M, Yu H, Li C (2018). Ultrasound Microbubble Delivery Targeting Intraplaque Neovascularization Inhibits Atherosclerotic Plaque in an APOE-deficient Mouse Model. In vivo.

[10] Yang H, Sun Y, Wei J, Xu L, Tang Y, Yang L (2019). The effects of ultrasound-targeted microbubble destruction (UTMD) carrying IL-8 monoclonal antibody on the inflammatory responses and stability of atherosclerotic plaques. Biomed Pharmacother.

[11] Su Y, Xu C, Li K, Wang B, Chen J, Liu L (2017). TGF-beta1 and TIMP1 double directional rAAV targeted by UTMD in atherosclerotic vulnerable plaque. Exp Ther Med.

[12] Sennoga CA, Kanbar E, Auboire L, Dujardin PA, Fouan D, Escoffre JM (2017). Microbubble-mediated ultrasound drug-delivery and therapeutic monitoring. Expert opinion on drug delivery.

[13] Rix A, Curaj A, Liehn E, Kiessling F (2019). Ultrasound Microbubbles for Diagnosis and Treatment of Cardiovascular Diseases.

[14] Liu Z, Gao S, Zhao Y, Li P, Liu J, Li P (2012). Disruption of tumor neovasculature by microbubble enhanced ultrasound: a potential new physical therapy of anti-angiogenesis. Ultrasound Med Biol.

[15] Li H, Lu Y, Sun Y, Chen G, Wang J, Wang S (2018). Diagnostic Ultrasound and Microbubbles Treatment Improves Outcomes of Coronary No-Reflow in Canine Models by Sonothrombolysis. Crit Care Med.

[16] Lu Y, Wang J, Huang R, Chen G, Zhong L, Shen S (2016). Microbubble-Mediated Sonothrombolysis Improves Outcome After Thrombotic Microembolism-Induced Acute Ischemic Stroke. Stroke.

[17] Wood AK, Bunte RM, Price HE, Deitz MS, Tsai JH, Lee WM (2008). The disruption of murine tumor neovasculature by low-intensity ultrasound-comparison between 1- and 3-MHz sonication frequencies. Acad Radiol.

[18] Wang J, Zhao Z, Shen S, Zhang C, Guo S, Lu Y (2015). Selective depletion of tumor neovasculature by microbubble destruction with appropriate ultrasound pressure. Int J Cancer.

[19] Michel JB, Virmani R, Arbustini E, Pasterkamp G (2011). Intraplaque haemorrhages as the trigger of plaque vulnerability. Eur Heart J.

[20] Moreno PR, Purushothaman KR, Fuster V, Echeverri D, Truszczynska H, Sharma SK (2004). Plaque neovascularization is increased in ruptured atherosclerotic lesions of human aorta: implications for plaque vulnerability. Circulation.

[21] ten Kate GL, Sijbrands EJ, Valkema R, ten Cate FJ, Feinstein SB, van der Steen AF (2010). Molecular imaging of inflammation and intraplaque vasa vasorum: a step forward to identification of vulnerable plaques?. J Nucl Cardiol.

[22] Shalhoub J, Monaco C, Owen DR, Gauthier T, Thapar A, Leen EL (2011). Late-phase contrast-enhanced ultrasound reflects biological features of instability in human carotid atherosclerosis. Stroke.

[23] Su T, Wang YB, Han D, Wang J, Qi S, Gao L (2017). Multimodality Imaging of Angiogenesis in a Rabbit Atherosclerotic Model by GEBP11 Peptide Targeted Nanoparticles. Theranostics.

[24] Xie J, Liao Y, Yang L, Wu J, Liu C, Xuan W (2011). Ultrasound molecular imaging of angiogenesis induced by mutant forms of hypoxia-inducible factor-1alpha. Cardiovasc Res.

[25] National Research Council Committee for the Update of the Guide for the C, Use of Laboratory A (2011). Guide for the Care and Use of Laboratory Animals.

[26] Shen S, Li Y, Xiao Y, Zhao Z, Zhang C, Wang J (2018). Folate-conjugated nanobubbles selectively target and kill cancer cells via ultrasound-triggered intracellular explosion. Biomaterials.

[27] Guo S, Shen S, Wang J, Wang H, Li M, Liu Y (2015). Detection of high-risk atherosclerotic plaques with ultrasound molecular imaging of glycoprotein IIb/IIIa receptor on activated platelets. Theranostics.

[28] Li X, Sun Y, Huang S, Chen Y, Chen X, Li M (2019). Inhibition of AZIN2-sv induces neovascularization and improves prognosis after myocardial infarction by blocking ubiquitin-dependent talin1 degradation and activating the Akt pathway. EBioMedicine.

[29] Li X, He X, Wang H, Li M, Huang S, Chen G (2018). Loss of AZIN2 splice variant facilitates endogenous cardiac regeneration. Cardiovasc Res.

[30] He X, Wang S, Li M, Zhong L, Zheng H, Sun Y (2019). Long noncoding RNA GAS5 induces abdominal aortic aneurysm formation by promoting smooth muscle apoptosis. Theranostics.

[31] Ay T, Havaux X, Van Camp G, Campanelli B, Gisellu G, Pasquet A (2001). Destruction of contrast microbubbles by ultrasound: effects on myocardial function, coronary perfusion pressure, and microvascular integrity. Circulation.

[32] van Hinsbergh VW, Eringa EC, Daemen MJ (2015). Neovascularization of the atherosclerotic plaque: interplay between atherosclerotic lesion, adventitia-derived microvessels and perivascular fat. Curr Opin Lipidol.

[33] Xu J, Lu X, Shi GP (2015). Vasa vasorum in atherosclerosis and clinical significance. Int J Mol Sci.

[34] Van der Donckt C, Van Herck JL, Schrijvers DM, Vanhoutte G, Verhoye M, Blockx I (2015). Elastin fragmentation in atherosclerotic mice leads to intraplaque neovascularization, plaque rupture, myocardial infarction, stroke, and sudden death. Eur Heart J.

[35] Potente M, Gerhardt H, Carmeliet P (2011). Basic and therapeutic aspects of angiogenesis. Cell.

[36] Mulligan-Kehoe MJ, Simons M (2014). Vasa vasorum in normal and diseased arteries. Circulation.

[37] Sluimer JC, Kolodgie FD, Bijnens AP, Maxfield K, Pacheco E, Kutys B (2009). Thin-walled microvessels in human coronary atherosclerotic plaques show incomplete endothelial junctions relevance of compromised structural integrity for intraplaque microvascular leakage. J Am Coll Cardiol.

[38] Pagiatakis C, Galaz R, Tardif JC, Mongrain R (2015). A comparison between the principal stress direction and collagen fiber orientation in coronary atherosclerotic plaque fibrous caps. Med Biol Eng Comput.

[39] Liang X, Xenos M, Alemu Y, Rambhia SH, Lavi I, Kornowski R (2013). Biomechanical factors in coronary vulnerable plaque risk of rupture: intravascular ultrasound-based patient-specific fluid-structure interaction studies. Coron Artery Dis.

[40] Virmani R, Kolodgie FD, Burke AP, Finn AV, Gold HK, Tulenko TN (2005). Atherosclerotic plaque progression and vulnerability to rupture: angiogenesis as a source of intraplaque hemorrhage. Arterioscler Thromb Vasc Biol.

[41] Van der Veken B, De Meyer GR, Martinet W (2016). Intraplaque neovascularization as a novel therapeutic target in advanced atherosclerosis. Expert Opin Ther Targets.

[42] Raj T, Kanellakis P, Pomilio G, Jennings G, Bobik A, Agrotis A (2006). Inhibition of fibroblast growth factor receptor signaling attenuates atherosclerosis in apolipoprotein E-deficient mice. Arterioscler Thromb Vasc Biol.

[43] Brown DL, Desai KK, Vakili BA, Nouneh C, Lee HM, Golub LM (2004). Clinical and biochemical results of the metalloproteinase inhibition with subantimicrobial doses of doxycycline to prevent acute coronary syndromes (MIDAS) pilot trial. Arterioscler Thromb Vasc Biol.

[44] Willems S, Vink A, Bot I, Quax PH, de Borst GJ, de Vries JP (2013). Mast cells in human carotid atherosclerotic plaques are associated with intraplaque microvessel density and the occurrence of future cardiovascular events. Eur Heart J.

[45] Taqueti VR, Di Carli MF, Jerosch-Herold M, Sukhova GK, Murthy VL, Folco EJ (2014). Increased microvascularization and vessel permeability associate with active inflammation in human atheromata. Circ Cardiovasc Imaging.

[46] Perrotta P, Emini Veseli B, Van der Veken B, Roth L, Martinet W, De Meyer GRY (2019). Pharmacological strategies to inhibit intra-plaque angiogenesis in atherosclerosis. Vascul Pharmacol.

