# Therapeutic ultrasound combined with microbubbles improves atherosclerotic plaque stability by selectively destroying the intraplaque neovasculature: Erratum

**DOI:** 10.7150/thno.81490

**Published:** 2023-04-17

**Authors:** Xinzhong Li, Shengcun Guo, Tong Xu, Xiang He, Yili Sun, Xiaoqiang Chen, Shiping Cao, Xiaoyun Si, Wangjun Liao, Yulin Liao, Yuan Han, Jianping Bin

**Affiliations:** 1 Department of Cardiology, State Key Laboratory of Organ Failure Research, Nanfang Hospital, Southern Medical University, Guangzhou, China.; 2 Guangzhou Regenerative Medicine and Health Guangdong Laboratory, 510005 Guangzhou, China.; 3 Department of Cardiology, The First Affiliated Hospital of Zhengzhou University, Zhengzhou, China.; 4 Department of Oncology, Nanfang Hospital, Southern Medical University, Guangzhou, China.

We regret that the immunohistochemistry images were misplaced and the mark of magnification position was missing in the Figure 3A and Figure 5A. The newly arranged Figure 3 and Figure 5 are shown below. Notably, the correction does not affect the conclusion of our paper. We genuinely apologize to the Editor and the readership of the journal for any inconvenience it may have caused.

## Figures and Tables

**Figure 3:**
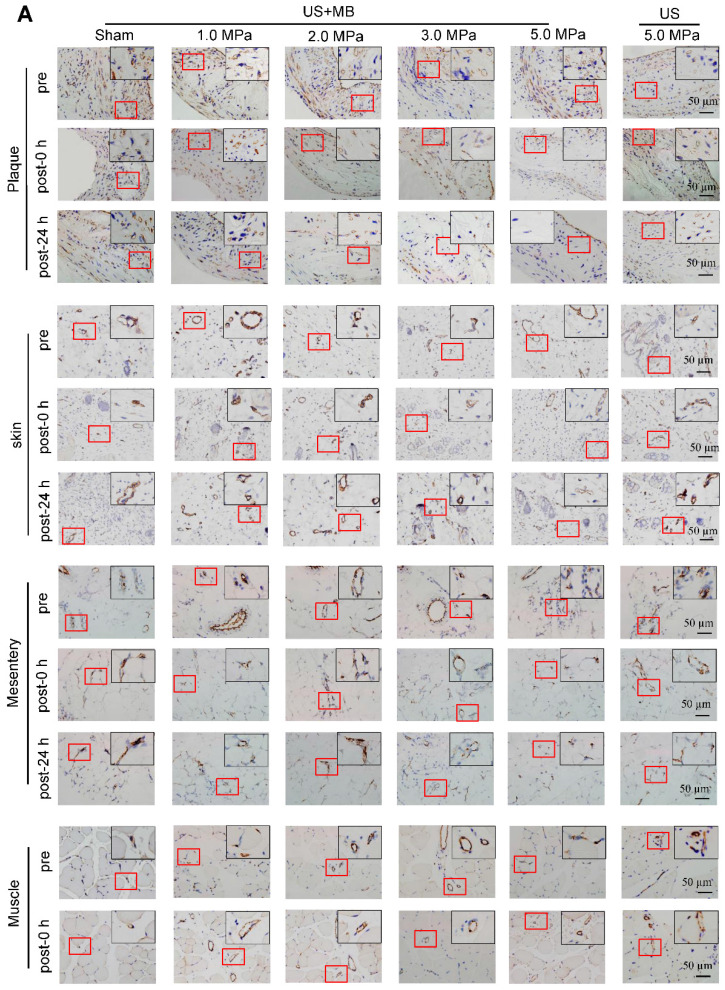

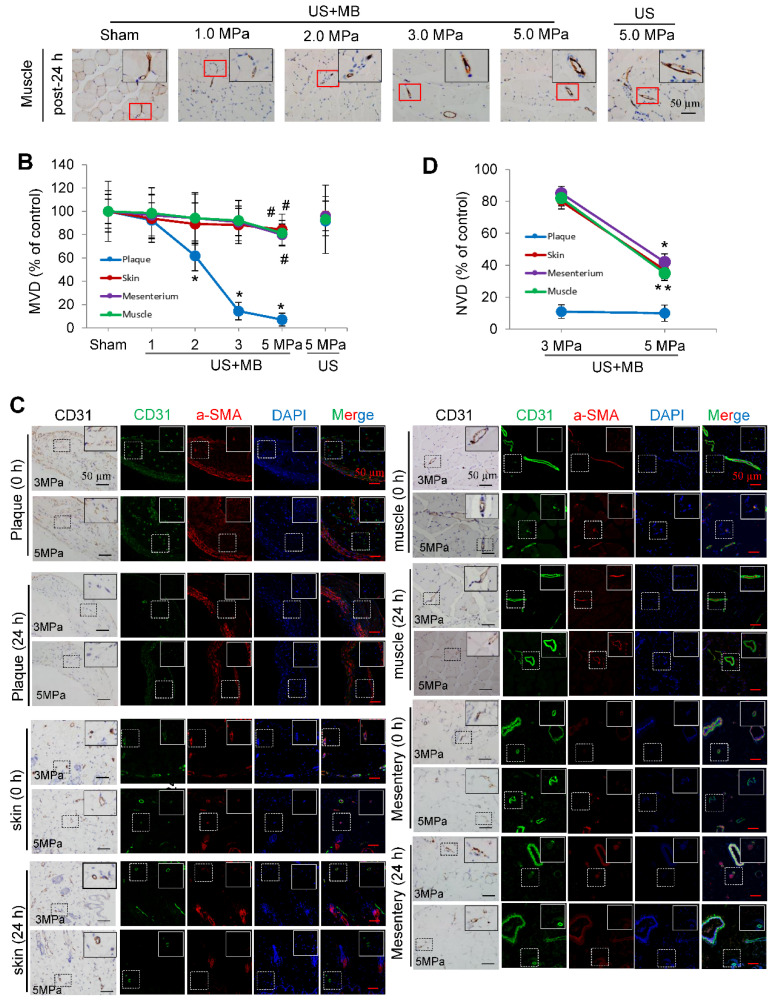
** Effects of US-MB treatment on microvessels in plaque, skin, mesentery and muscle: Results for various pressures.** (A) Representative images of immunohistochemical staining for the endothelial marker CD31 in plaque, skin, mesentery and muscle (bars, 50 μm). (B) Quantitative analysis of the MVD at 24 h after treatment. *p < 0.05, ^#^p < 0.05, vs. the respective sham groups. MVD, microvessel density. (C) Representative images of immunohistochemical staining for CD31 and confocal immunofluorescence of microvessels in plaque, skin, mesentery and muscle stained for CD31 (green) and α-SMA (red) at 0 h and 24 h after treatment (bars, 50 μm). (D) Quantitative analysis of neovessels. *p < 0.05 vs. post 0 h. SMA, smooth muscle actin.

**Figure 5:**
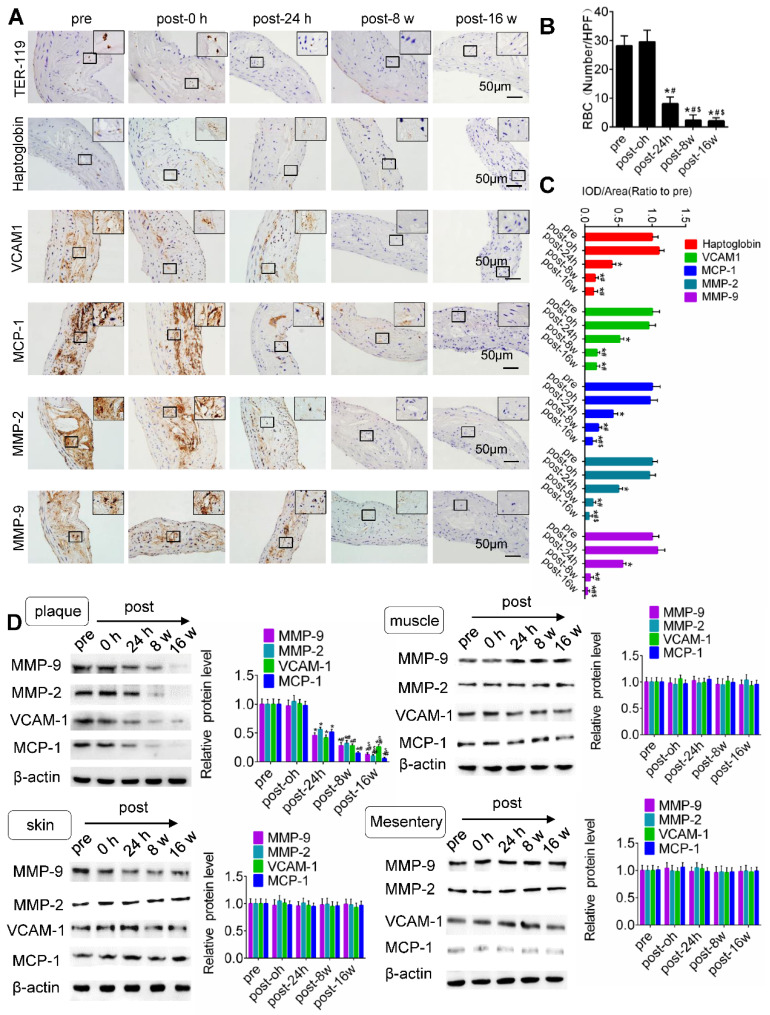
** TUS-MB treatment reduced intraplaque extravasation of erythrocytes and secondary inflammation.** (A) Representative images of immunohistochemical staining for TER-119, hemoglobin, VCAM1, MCP-1 and MMP-2 pretreatment and at 0 h, 24 h and 8 weeks after treatment with TUS-MB at 3.0 MPa (bars, 50 μm). (B) Quantification of the average number of red blood cells. *p < 0.05 vs. pre. ^#^p < 0.05 vs. post-24 h; n = 6 per group. (C) Quantification of the expression of hemoglobin, VCAM1, MCP-1 and MMP-2 at different time points. (D) Representative immunoprecipitation images of VCAM1, MCP-1, MMP-2 and MMP-9 in plaque, skin, muscle and mesentery pretreatment and at 0 h, 24 h, 8 w and 16 w after treatment with TUS-MB at 3.0 MPa (bars, 50 μm). *p < 0.05 vs. pretreatment. ^#^p < 0.05 vs. 24 h posttreatment; n = 6 per group.

