# Supplementary material for: Therapeutic ultrasound combined with microbubbles improves atherosclerotic plaque stability by selectively destroying the intraplaque neovasculature
Source: Theranostics. 2020 Jan 22;10(6):2522–37. doi: 10.7150/thno.39553 (PMC7052908; doi:10.7150/thno.39553)
Supplement: Supplementary file 1 — Supplementary figures. [file thnov10p2522s1.pdf]

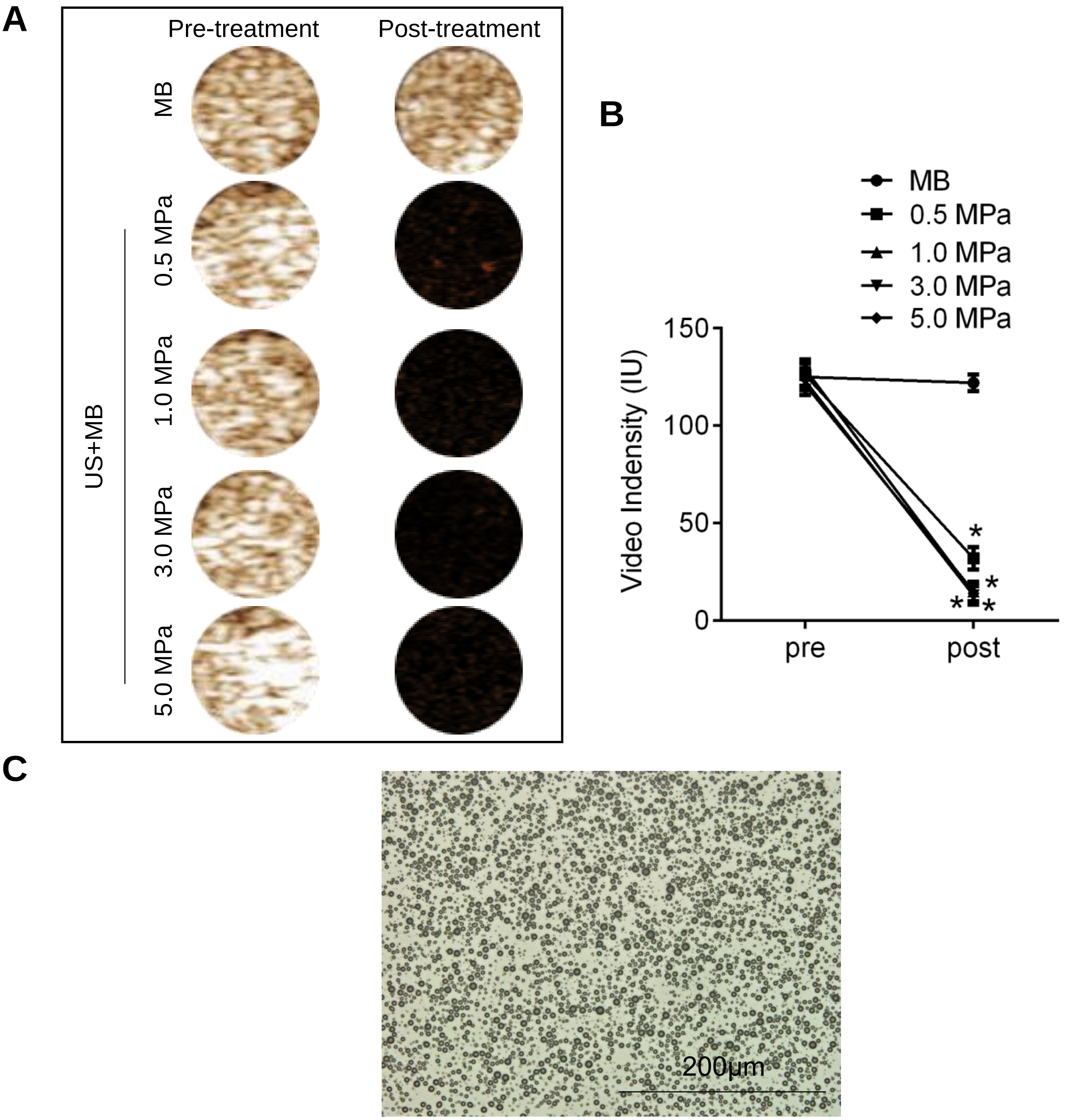

D

	Concentration（10^7/ml）	Mean diameter(μm)
microbubbles	8.45±1.18	2.41±1.21

Physical characteristics of microbubbles. (A) Contrast-enhanced ultrasound (CEUS) images of MBs loaded in the water at 37°C before and after treatment with different ultrasonic pressure or no ultrasound. (B) Quantitative analysis of the video intensity of CEUS images. *p < 0.05 vs. pre. n =6 per group. (C, D) Graph of the size distribution and concentration of microbubbles (MBs) (bar 200μm).

Figure S1

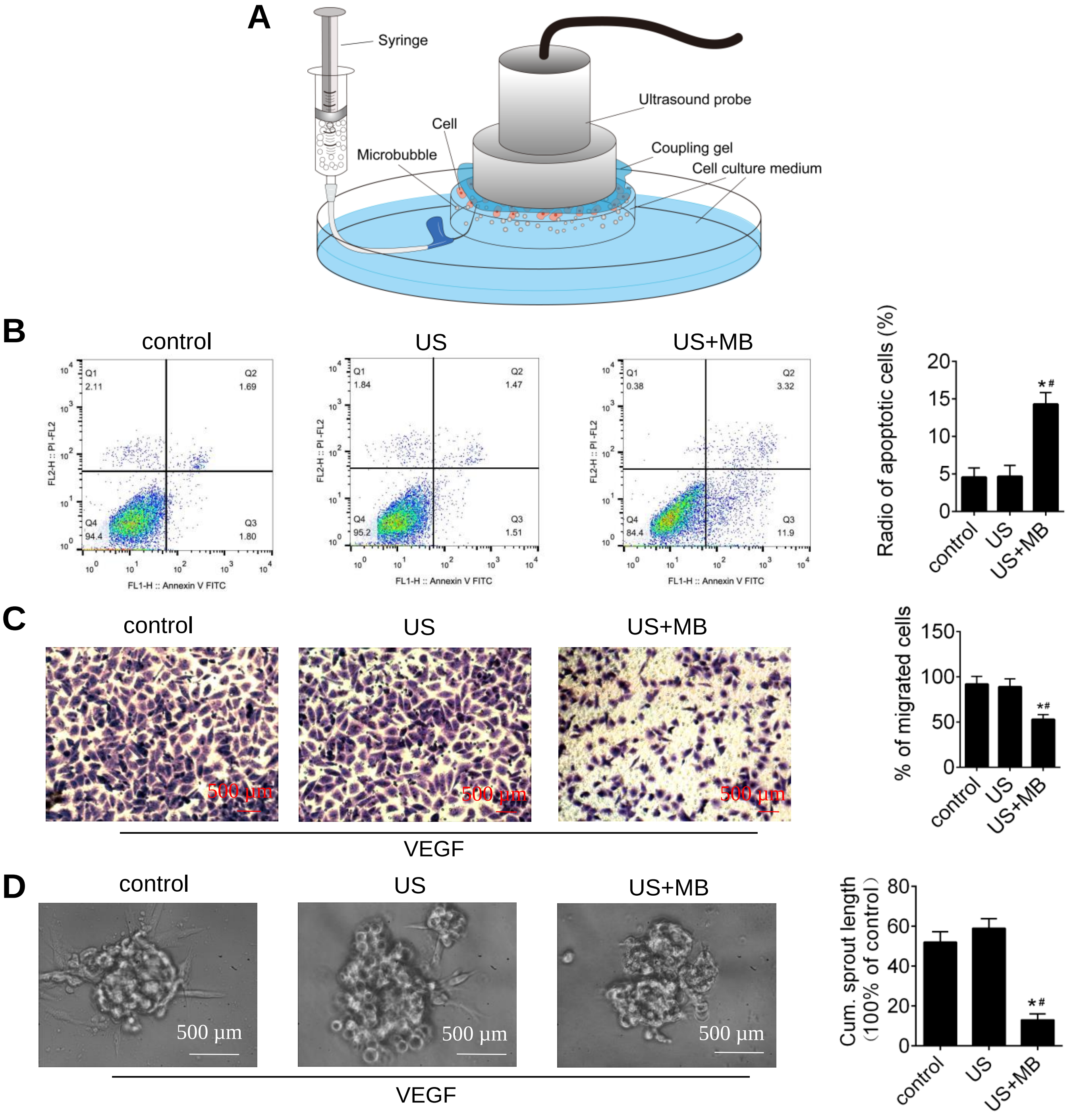

(A) Illustration of *in vitro* experimental design. (B) Flow cytometric analysis of HUVEC apoptosis was performed twenty-four hours treated by LIUS-MB with 1.0 MPa. $^{*}p<0.05$ vs. control. $^{\#}p<0.05$ vs. US; n=6 per group. (C) HUVEC invasion assessed by transwell assay (bars, 500 μm). $^{*}p<0.05$ vs. control. $^{\#}p<0.05$ vs. US; n=6 per group. (D) Representative images of vascular sprouting. HUVEC spheroids were allowed to undergo sprouting in a 3-dimensional (3D) matrix twenty-four hours treated by VEGF (bars, 500 μm). $^{*}p<0.05$ vs. control. $^{\#}p<0.05$ vs. US; n=6 per group. VEGF (10 ng/mL) was used to induced endothelial invasion and sprouting.

Figure S2

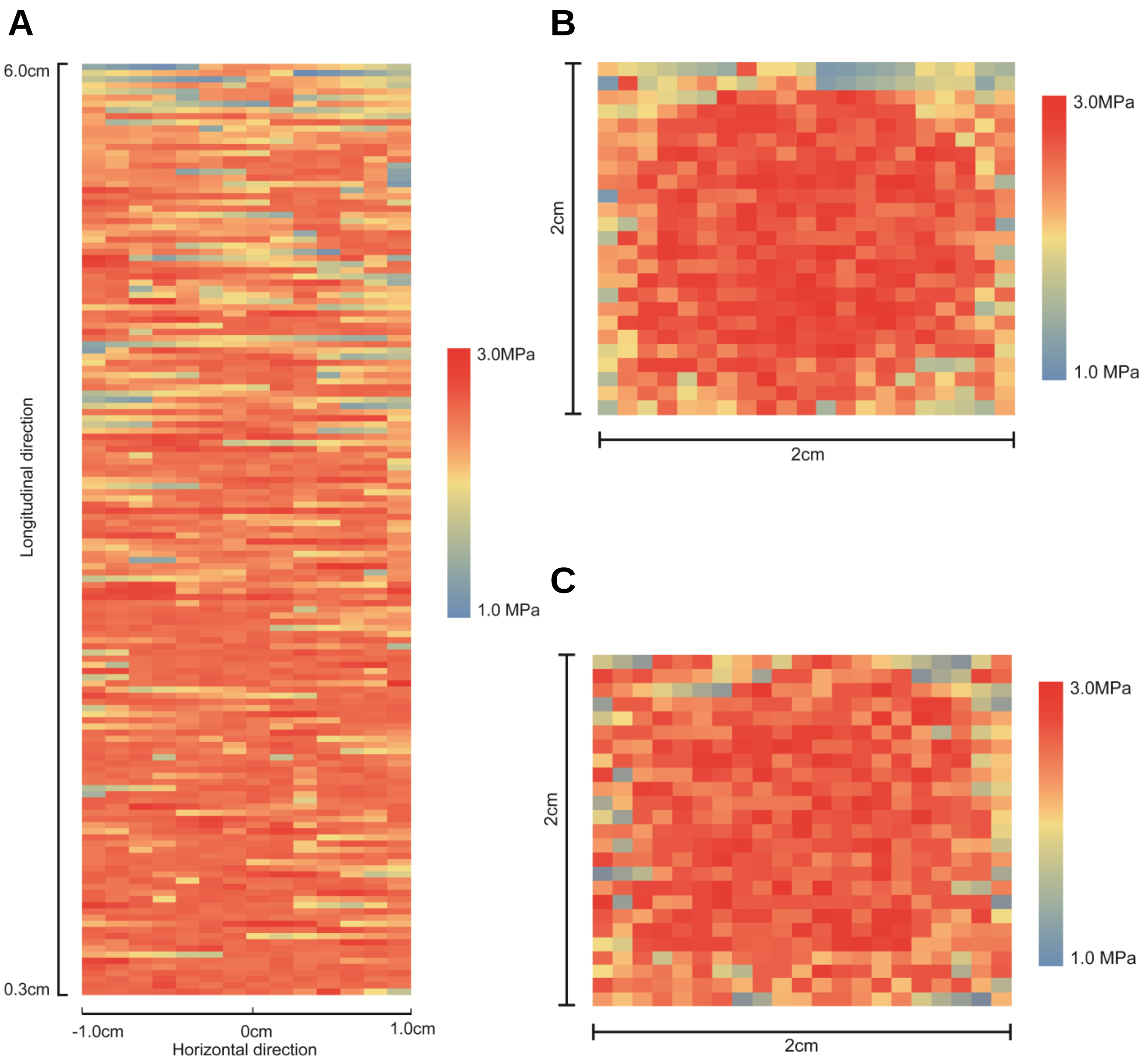

Heat map display of ultrasonic transducer on the vertical and horizontal axes. The spectrum spans fold changes from 1.0 MPa to 3.0 MPa. (A) The sound pressure from 0.3 cm to 6.0 cm from the transducer is shown on the vertical axis. The sound pressure on the horizontal axis shows the cross section at 1.0 cm (B) and 2.0 cm (C) from the transducer.

Figure S3

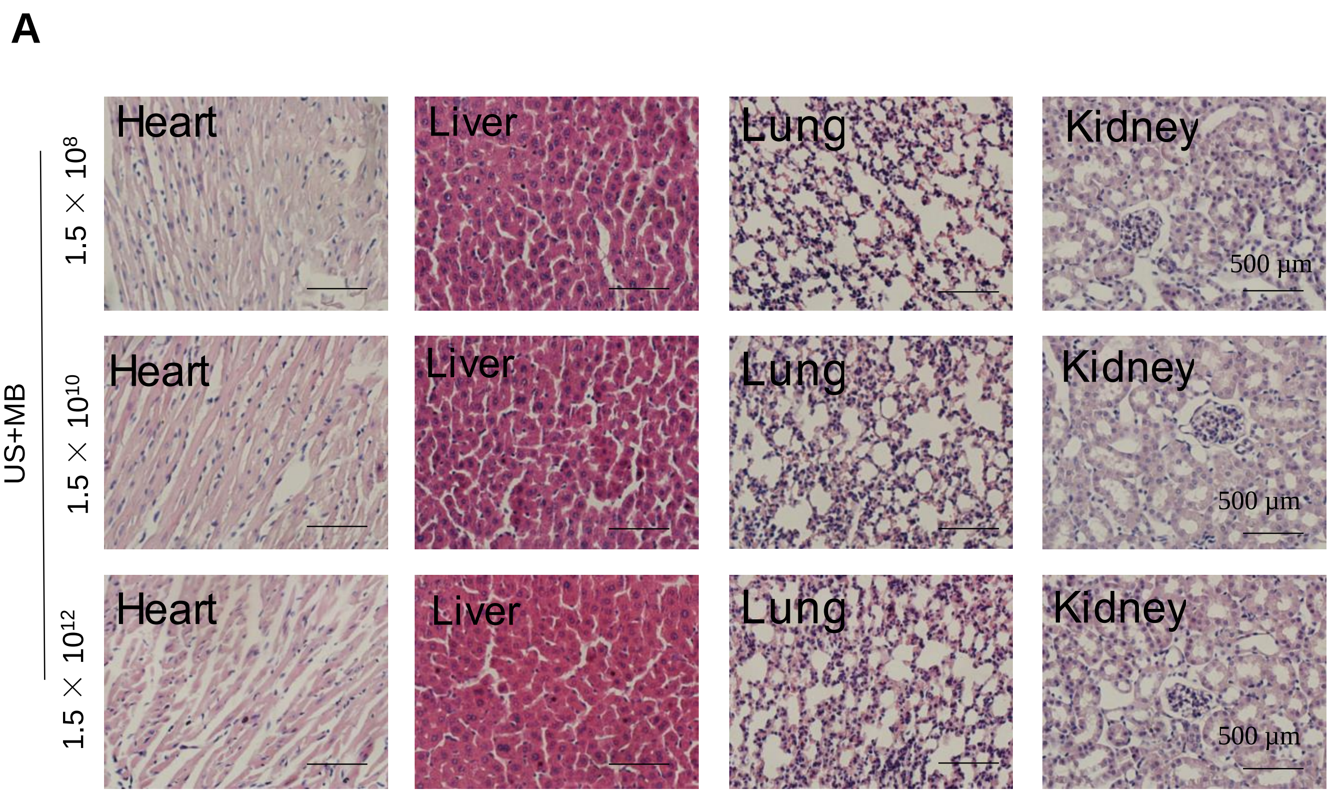

(A) Representative hematoxylin and eosin staining of tissue samples from mice after US-MB treatment with 3.0 MPa at different MB concentrations (bar 500μm). The stained images show no obvious injury or necrosis (i.e., few inflammatory cells, such as neutrophils, and apoptotic cells and little swelling). US: ultrasound. MB: microbubble.

Figure S4

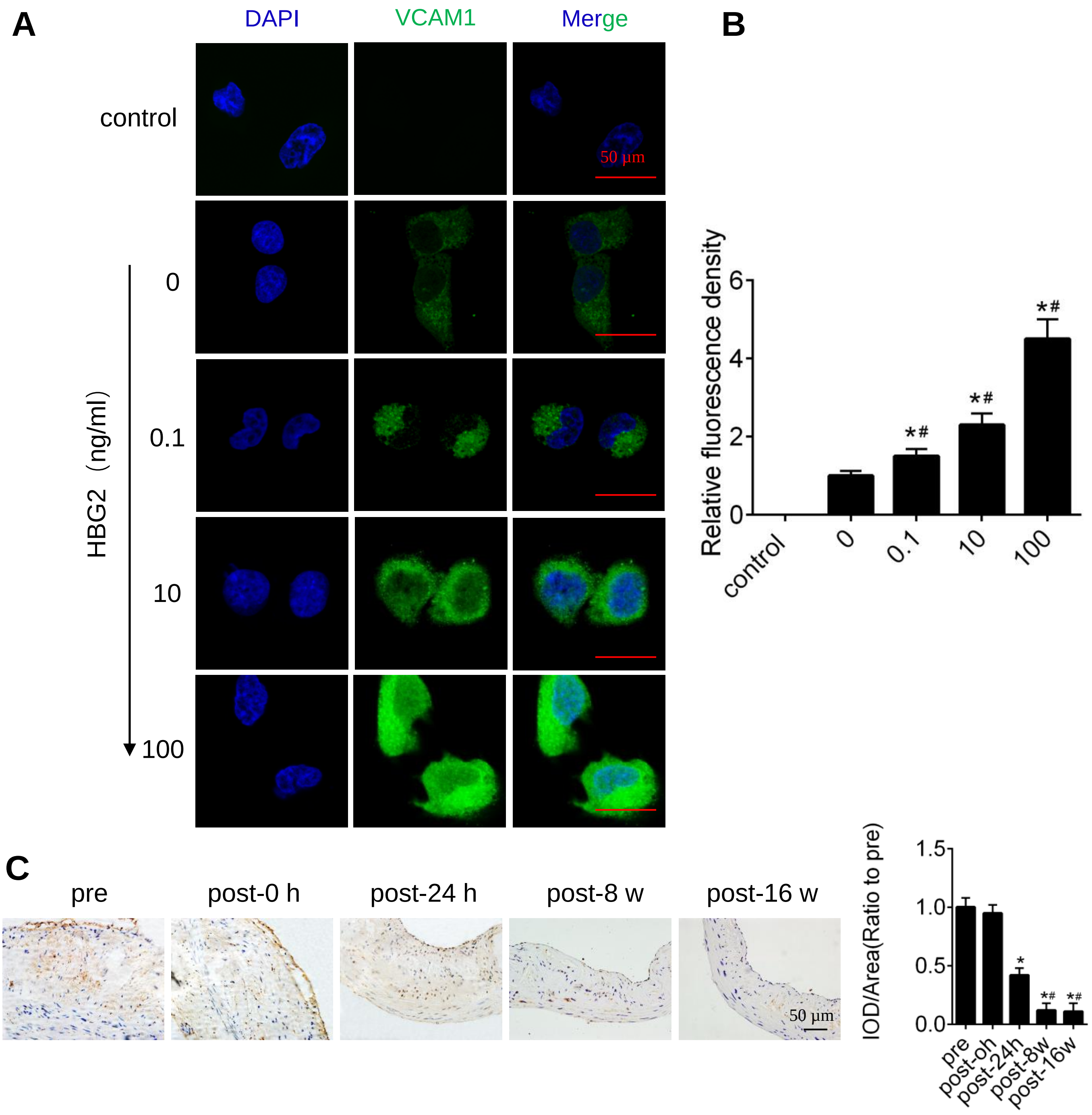

(A) Representative confocal images of HUVECs stimulated with various concentrations of HBG2 (Recombinant Human Hemoglobin Gamma G) (bars, 50 μm). (B) Quantitative analysis of relative fluorescence signal intensity of VCAM1. $^{*}p<0.05$ vs. control. $^{\#}p<0.05$ vs. HBG2 (0 ng/ml); n=6 per group. (C) Representative images of immunohistochemical staining for CD41 (bars, 50 μm). $^{*}p<0.05$ vs. pre. $^{\#}p<0.05$ vs. post-24h; n=6 per group.

Figure S5
